# Ameloblastoma giant: Diagnosis, treatment and reconstruction: A case report

**DOI:** 10.1016/j.amsu.2021.102589

**Published:** 2021-07-26

**Authors:** Sanaa ELmrini, Mohamed Raiteb, bahaa razem, Fatema zahra Azami Hassani, Faiçal Slimani

**Affiliations:** a Faculty of Medicine and Pharmacy, Hassan II University of Casablanca, B.P, 5696, Casablanca, Morocco; b Oral and Maxillofacial Surgery Department, CHU Ibn Rochd, B.P, 2698, Casablanca, Morocco

**Keywords:** Ameloblastoma, Mandibular, Tumors, Surgery, Case report

## Abstract

**Introduction:**

Ameloblastoma is a rare tumor, benign but rapidly extensive and prone to recurrence. Its management remains difficult and its treatment relies mainly on surgery. For giant ameloblastoma or in people with an advanced stage the gesture remains very mutilating.

**Presentation of case:**

This is a 22 year old patient, the onset of the symptomatology dates back to 2 years ago with the appearance of a mandibular swelling that increased in size with dental mobility. this motivated the patient to consult a CT scan and a panoramic radiograph as well as a biopsy that objectified an ameloblastoma. He benefited from a surgical excision with reconstruction.

**Discussion:**

It is a rare tumor that mainly affects young people and especially males. It can be discovered by chance or generally in front of a mandibular swelling. Radiological examination is essential as well as biopsy to confirm the diagnosis. The treatment is surgical, which consists of an exeresis with safety margins. Reconstruction should be discussed especially for young healthy subjects.

**Conclusion:**

Although ameloblastoma remains a benign tumor, it is a tumor that evolves rapidly and recurs a lot, which is why it is necessary to take care of it quickly with a radical treatment and a regular follow-up with the patients.

## Highlights

• Ameloblastoma is a rare benign but locally aggressive tumor that recurs frequently.
• Ameloblastoma remains a benign tumor whose management must be rapid to avoid significant loss of substance because the choice of reconstruction remains difficult.
• The maxillofacial surgeon must choose between different treatments with a regular follow-up of the patient.

## 1 Introduction

**Fig. 1:**
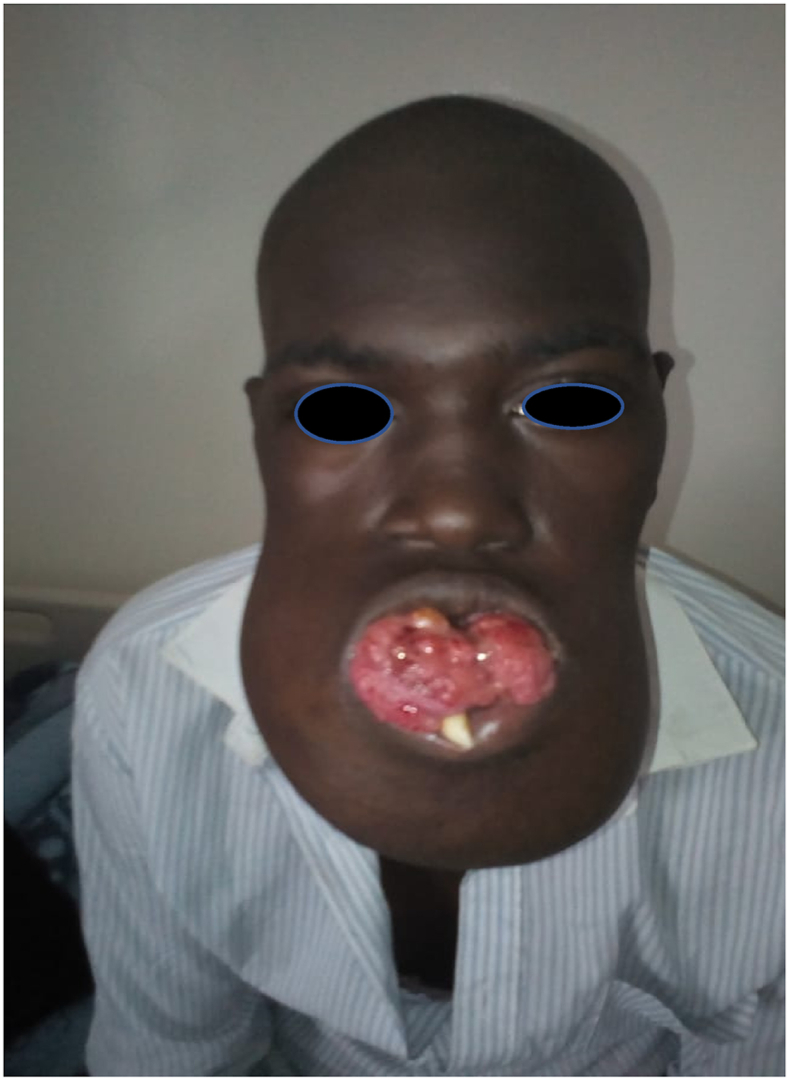
Giant ameloblastoma.

**Fig. 2:**
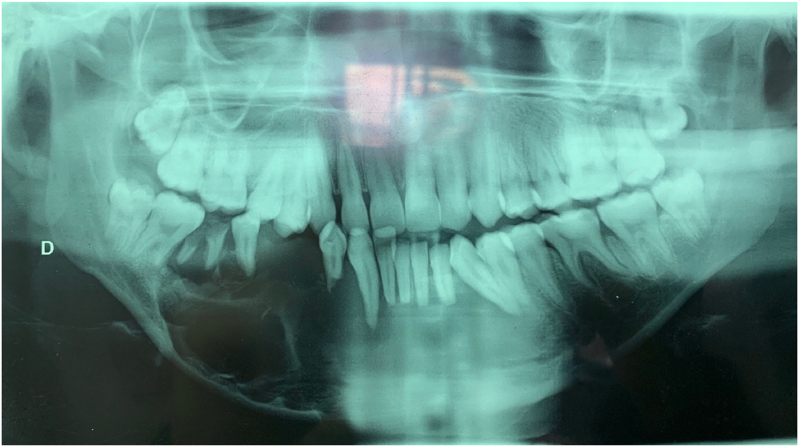
The panoramic which objectifies a multilocular image.

**Fig. 3:**
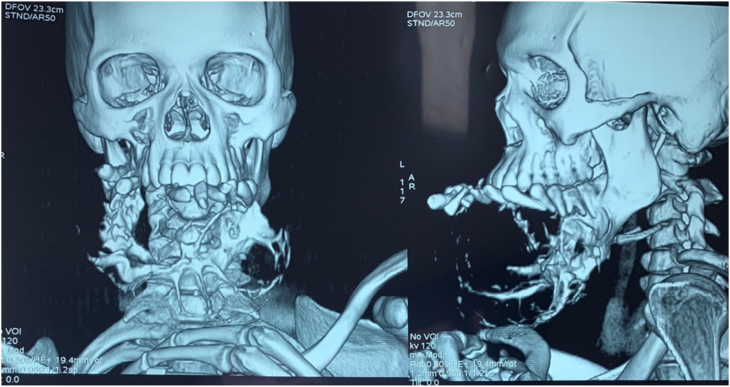
3D reconstruction that shows us the extent of the tumor.

**Fig. 4:**
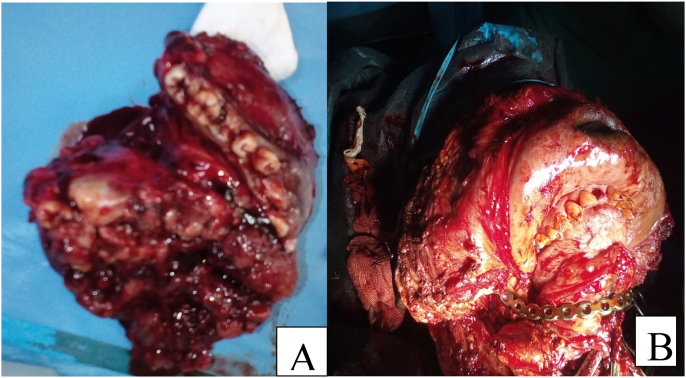
**A:** Exceresis piece tumor**B:** Reconstruction with maxi plate.

**Fig. 5:**
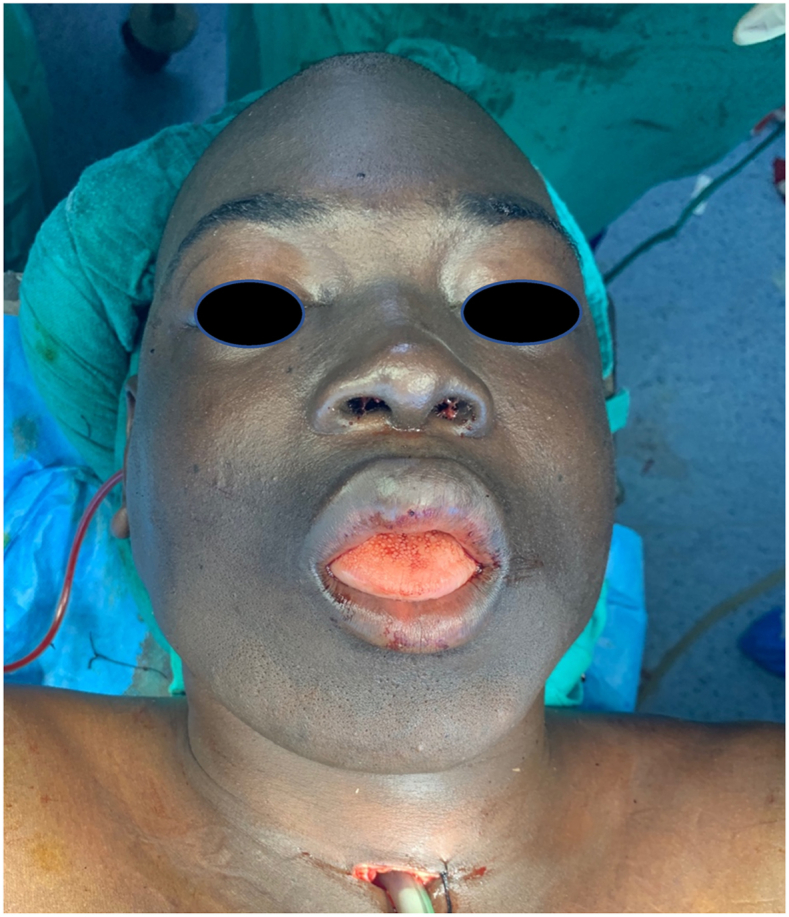
Postoperative patient after excision and reconstruction.

Ameloblastoma is a benign tumor. It is an epithelial neoplasm developed from dento-forming cells without a mesenchymal component [1] (see Fig. 1, Fig. 2, Fig. 3, Fig. 4, Fig. 5)

Clinically ameloblastoma manifests as a jugal or symphyseal swelling all depends on the location. The diagnosis is made by panoramic radiography and CT scan. The confirmation is done by anatomopathological examination. Management must be rapid so that the procedure is as non-mutilating as possible. Regular monitoring is necessary to avoid any recurrence.

L'a^ge médian de survenue est de 35 ans, les deux sexes sont également affectés, avec une prédominance chez la race noire pour certaines études. La majorité des améloblastomes sont polykystique et sont plus difficiles à éradiquer que les variétés mono-kystiques et périphériques [2].

Ces ame ´loblastomes se divisent en ame ´loblasto-mes intra-osseux (centraux) et tissulaires (pe ´riphe ´riques). Il existe, par ailleurs, diffe ´rents types histologiques d'ame ´lo-blastomes (folliculaires, kystiques, unikystiques, plexiformes et mixtes …)

Nous allons presenté un cas d'ameloblastome dont le diagnostic sest fait tardivement et donc la chirurgie a été mutilante d’ ou linteret d'une prise en charge rapide.

This case report has been reported in line with the SCARE Criteria(2) [3].

## 2 Case report

The patient was 22 years old with no particular pathological history. The onset of the symptomatology dates back to 2 years ago with the appearance of a painful mandibular swelling of the mandible progressively increasing in volume with dental mobility, which motivated the patient to consult (Fig. 1). On clinical examination, we found a swelling of the body of the mandible that bled on contact with the teeth. The swelling respects the skin but infiltrates the lower vestibule and the floor of the mouth, pushing the tongue backwards. A biopsy was performed which showed a follicular and plexiform ameloblastoma. A CT scan showed a large polycystic mass with extensive bone lysis along the body of the mandible, which came into contact with the soft tissue without infiltration (Fig. 2, Fig. 3). The surgical intervention consisted of a total and radical resection of the tumor. A resection of the mandibular body was performed with the placement of a maxi plate. The postoperative course was simple (Fig. 4, Fig. 5).

The patient was seen at the consultation with good improvement and good healing. The anatomopathological examination confirmed the nature of a follicular and plexiform ameloblastoma without any sign of malignancy.

## 3 Discussion

Ameloblastoma is a benign tumor of odontogenic origin, locally aggressive but slowly progressive. Its origin is the epithelium involved in tooth formation, namely the enamel organ, the epithelial remnants of Malassez, the wall of odontogenic cysts (dentigers) or also the basal epithelial cells of the oral mucosa [4].

Ameloblastoma are rare and represent only 1% of all maxillary tumors and 11% of odontogenic tumors [5].

The etiology is of unknown origin; so far, no potential risk factors have been identified.

Ameloblastoma affects with predilection the male sex, it seems that the disease is more frequent in black subjects [6] as it is the case of our patient.

Ameloblastoma most often involves the mandibular more than 80% [1] the angular region represents the most frequent location, with extension to the horizontal branch (70%), then come the para symphyseal (20%) and symphyseal (10%) regions. The tuberosity constitutes the preferential maxillary location [1].

Ameloblastoma is characterized by a slow but rapidly extensive evolution which makes the management difficult and the surgical procedure very mutilating.

The most frequent reason for consultation is facial swelling, whether mandibular or maxillary.

The delay in consultation can be explained either by the slow evolution of the tumor, the negligence due to the fact that the tumors are painless and of slow evolution let think that the process will regress spontaneously, the low socio-economic level and the geographical distance of the patients [7].

Most of the time, ameloblastoma are discovered by chance, by radiography made for other reasons.

Ameloblastoma has a general histologic structure made up of a center formed by islands that have radiated epithelial cells with a loose texture with frequent formation of microcysts, and a periphery consisting of an epithelium whose cells have an inverted nuclear polarity and supra nuclear vacuoles [8].

There are many histological types: follicular, uni-cystic, plexiform, peripheral, desmoplastic.

The radiological examination is essential for the diagnosis, it allows us to orientate ourselves but only the anatomopathological examination allows to confirm it.

Several types of images can be found at the radiological level:

A unilocular cystic image that can be confusing.

A multilocular image that translates into a honeycomb or soap bubble image.

### 3.1 A vast and flawed picture

In the event of a delay in diagnosis, do not hesitate to request a scan in addition to an orthopantomogram.

On scan, it is usually an extensive tumor surrounded by a bony shell, with irregular boundaries with hypo dense cystic areas and solid iso dense areas with contrast of the bony component after injection [1].

The 3D reconstruction gives us information on the limits of the tumor and its relationship with the lower dental canal.

The IRM is indicated when the tumor arrives at the level of the soft parts.

Imaging is used to guide the diagnosis and follow-up of ameloblastoma.

Ameloblastoma recurs frequently, so a radical treatment is necessary. There are two types of treatment: conservative treatment by marsupialization, enucleation and curettage, and radical treatment which consists of resection of the bone tissue while respecting the margins of exeresis.

The treatment is surgical, it is generally mutilating especially for advanced cases. The surgical treatment can be conservative or radical, the therapeutic decision must take into consideration several factors including the age of the patient, the anatomical location of the lesion, its extension, the radiological aspect, the evolutionary potential and the probabilitý of a regular follow-up of the patient [1].

Even after apparently well-conducted surgical treatment and despité wide resections with pathologically healthy recuts, ameloblastoma is likely to recur probably because of micro tumor bone foci existing distant from the main tumor [9].

Many authors advocate a wide resection at the outset with a safety margin in the periphery [10].

## 4 Conclusion

Ameloblastoma is a rare tumor, benign but locally very aggressive, its treatment is mainly surgical. The surgical gesture can be very mutilating especially when the diagnosis is made late, hence the interest of a rapid management with a radical surgical treatment and limits of wide exeresis to avoid as much as possible a recurrence.

Proton beam irradiation would likely be advantageous for patients with maxillary ameloblastomas extending to the skull base to adequately treat the tumdor and reduce the dose to the central nervous system and visual system [11].
